# Understanding erosion resistance mechanisms of sodium aluminate silicate hydrate in erosion environments: a molecular dynamics study

**DOI:** 10.1039/d4ra00302k

**Published:** 2024-04-02

**Authors:** Qingyin Tang, Mengqi Sun, Xinghai Lu, Dongshuai Hou, Mengmeng Li, Pan Wang

**Affiliations:** a Department of Civil Engineering, Qingdao University of Technology Qingdao 266033 China limengmeng7@126.com

## Abstract

Sodium-aluminate-silicate-hydrate (NASH) gel, as the primary reaction product stimulated by alkali in silica-aluminum-rich minerals, influences the mechanical and durability properties of geopolymers. In erosion environments, NASH demonstrates superior compressive strength and erosion resistance compared to hydration products of ordinary Portland cement. However, the underlying erosion resistance mechanism of NASH under such conditions remains unclear. Therefore, this study employs molecular dynamics research methodology to investigate the alteration in performance and deterioration mechanism of NASH in erosive environments. The findings reveal that in Na_2_SO_4_ solution, the infiltration of H_2_O molecules and Na^+^ ions into the three-dimensional mesh structure of NASH results in slight expansion and reduced tensile strength. Although H_2_O intrusion induces hydrolysis of the three-dimensional skeleton, the adsorption sites within NASH possess the capability to capture externally introduced Na^+^ ions. During tensile loading, Na^+^ ions can interact with reactive oxygen species produced through stretching or H_2_O molecule-induced decomposition of the internal framework, facilitating the repair of fractured structures. Consequently, this process partially alleviates tensile rupture, modifies the fracture damage mode, enhances overall toughness, and improves resistance against sulfate attack.

## Abstract 2

Sodium-aluminate-silicate-hydrate (NASH) gel, as the primary reaction product stimulated by alkali in silica-aluminum-rich minerals, influences the mechanical and durability properties of geopolymers.

## Graphical abstract

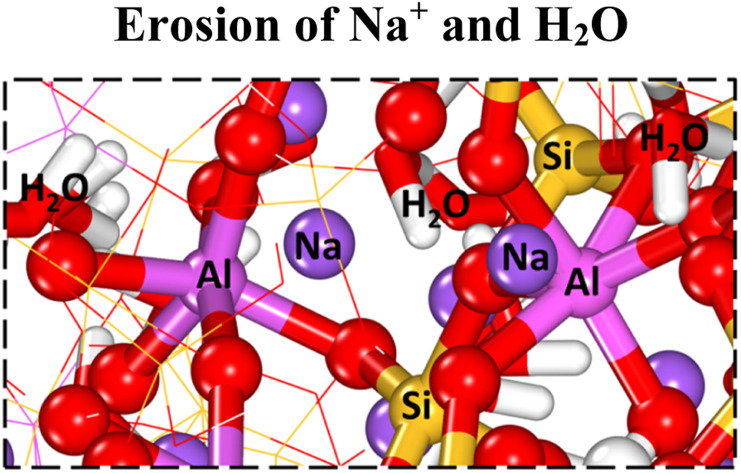

## 1 Introduction

In recent years, the issue of carbon dioxide emissions has received international attention. Silicate cement material is the backbone of infrastructure and is also a high-carbon emission material.^1^ Research and development of low energy consumption and environmentally friendly building materials is urgent. Geopolymers are known as sustainable green cementitious materials due to their low energy consumption and low CO_2_ emissions during production, and the interest in Geopolymer Cementitious Materials (GCM)^2^ has been increasing year by year. Compared with Ordinary Silicate Cement Materials (OSCM), the geopolymer gel structure has higher strength and better stability,^3^ and has superior chemical resistance in acid, alkali and corrosive salt environments.^4,5^ Notably, geopolymer preparation does not rely on conventional cement but utilizes recycled waste (fly ash, slag, *etc.*), which can reduce the necessity for limestone calcination and carbon emissions,^6^ enabling efficient waste resource utilization, lessening environmental impact, diminishing reliance on natural resources, and providing enhanced environmental benefits. In addition, common silicate cement structures in marine environments are subjected to a variety of aggressive influences such as elevated temperatures, high salinity,^7^ and cyclical wet and dry conditions.^8^ These factors impose a significant risk of degradation or even structural failure on cement-based materials throughout prolonged service periods. Research has demonstrated that the presence of Cl^−^ and SO_4_^2−^ ions in seawater is a crucial factor leading to extensive deterioration in both the mechanical properties and durability of marine concrete.^9,10^ Concrete exposed to a sulfate-laden environment results in the infiltration of sulfates into the internal matrix *via* the concrete's pore system, inducing the formation of expansive reaction by-products. This process culminates in mechanical manifestations, such as cracking and spalling, adversely impacting the structural integrity of the concrete.

To address the issue of insufficient durability of marine concrete and the problem of environmental pollution, current engineering practices commonly employ several methods to enhance the concrete's permeability, such as polymer-modified concrete,^11^ cathodic protection,^12^ epoxy coatings,^13^ surface coatings, and the incorporation of rust inhibitors.^14^ While these methods exhibit a certain level of maturity, further research on erosion-resistant materials is required, considering the current global challenges of resource scarcity and environmental pollution.^15^ Geopolymers as a green material, has been gradually focused on its application prospects in marine engineering. Geopolymers,^2,16^ characterized by a zeolite-like cage structure, present superior mechanical properties, excellent thermal stability, and enhanced durability when compared to Portland cement, which possesses a layered structure. As a novel environmentally friendly binder, geopolymer has found extensive application in various fields, including construction, wastewater treatment, and aerospace materials.

Currently, some researchers have conducted relevant alkali excitation experiments and performance tests on geopolymers in erosive environments by experimental means, and found the structural and performance differences between geopolymers and ordinary silicate-containing materials. The inclusion of materials like blast furnace slag results in the formation of low-calcium calcium-aluminate-silicate-hydrated (CASH) gel, which exhibits a layered structure similar to calcium-silicate-hydrate (CSH).^17^ Incorporating materials such as fly ash, leads to the formation of alkaline NASH gel with a unique three-dimensional network structure due to its higher aluminum content.^18^ Experimental preparation of geopolymers typically involves the use of alkali activators. Ismail *et al.*^19^ explored the effects of alkali activators on the evolution of fly ash/slag (1 : 1) in a sulfate environment. They discovered that the sulfate itself had no direct impact on the erosion of GPC; instead, the nature of cations in the solution strongly influenced the evolution process. When GPC was immersed in Na_2_SO_4_ solution, binder degradation into sulfate precipitates did not occur due to the absence of a calcium (Ca) phase in the NASH matrix, enabling NASH to maintain stable existence and development in Na_2_SO_4_ solution.^20^ Bakharev *et al.*^21^ exposed alkali slag and ordinary silicate cement to 5% Na_2_SO_4_ and Mg_2_SO_4_ solutions, respectively. They observed that alkali slag displayed greater resistance to compressive strength reduction in Na_2_SO_4_ solution. This is because of the cage-like structure of the GPC, which prevents the intrusion of harmful ions, and the reduction in calcium content, which weakens the swelling of degradation products. Kumar *et al.*^22^ explored the microstructural features of alkali-activated geopolymers, uncovering a significant increase in compressive strength compared to OPC. The enhancement was linked to the more compact microstructure of geopolymers and the development of extra crystalline phases.

Much previous research has primarily focused on macroscopic experimental investigations into the degradation properties of geopolymers in erosive environments. In recent years, the feasibility and potential benefits of studying the microstructure of materials and gaining nanoscale insights into various interactions through molecular dynamics (MD) simulations have garnered widespread recognition among scholars. MD simulation methods offer an effective approach for revealing atomic-level details. MD simulations have found extensive use in investigating NASH, encompassing the polymerization formation process, fracture toughness analysis, and examination of adsorbed heavy metal ion properties.^23,24^ Guan *et al.*^25^ presented a detailed construction process of NASH with different Al/Si, which helped us to systematically construct NASH for molecular simulation studies. Hou *et al.*^26^ simulated the condensation reaction process of NASH, which provided more detailed nano-insights for constructing NASH models. Zhang *et al.*^18^ studied the impact of structural H_2_O on NASH's microstructure. Their findings revealed that the hydrolysis reaction induced by H_2_O molecules altered the backbone structure, leading to elongation of the Al–O bond, reduced stability, increased presence of five-coordinated aluminum, and disruption of the aluminate backbone. Consequently, this resulted in a decline in the material's mechanical properties. In NASH not only the structural H_2_O but also the Ca content inside the matrix affects the mechanical properties, Wang *et al.*^27^ investigated this and found that the more Ca content, the stronger the mechanical properties of NASH. Not only internal factors but also external factors affect the structural stability of NASH, especially concrete serving in the sea is more eroded by external solutions. Zhang *et al.*^28^ also examined the adsorption behavior of Na_2_SO_4_ and MgSO_4_ solutions on NASH's surface. They observed that the disparity in adsorption primarily depended on the cations present in the external solution. The binding of Mg^2+^ ions to the substrate was more stable than that of Na^+^ ions and Mg-containing precipitates were observed. Currently, research is scarce on the properties and mechanisms of the external environment on NASH cementitious materials, especially the microscopic mechanism of salt solution on the substrate is not clear. The reaction mechanism underlying the effects of sulfate on the mechanical properties of these binders requires further in-depth investigation.

This study investigates the mechanical properties and reaction mechanism of NASH gelling materials under an erosion environment. Through the reaction force field construction and simulation of NASH, the gel-solution coexistence system was constructed from the molecular level, and the interaction between the gel and the external solution was explored. The tensile properties of NASH were subsequently examined under vacuum, H_2_O, and sodium sulfate environments. Furthermore, through a comprehensive analysis, the underlying micro-mechanisms responsible for the observed changes in performance were elucidated. The findings of this study provide valuable insights into achieving a broader application of geopolymers as a replacement for silicate cement.

## 2 Simulation methods

### 2.1 Reactive force filed

In this study, the degradation properties of geopolymers in various environments will be simulated using the Reactive Force Field (ReaxFF).^29^ ReaxFF is a molecular dynamics force field specifically designed for complex systems, offering enhanced computational efficiency and reduced storage requirements. In comparison to conventional force fields, ReaxFF enables more accurate modeling of chemical reactions, encompassing processes such as bond formation and cleavage. The ReaxFF parameters^29–31^ for different elements have been derived from previously published studies and are widely utilized in investigations concerning the H_2_O dissociation of CSH gels,^32,33^ polymerization reactions in polymer systems, and the conformational and kinetic behavior of polymer chains. Employing ReaxFF simulations offers valuable insights into polymer properties, structural changes, and optimization of polymerization processes, among other applications.

### 2.2 Model construction

**Fig. 1:**
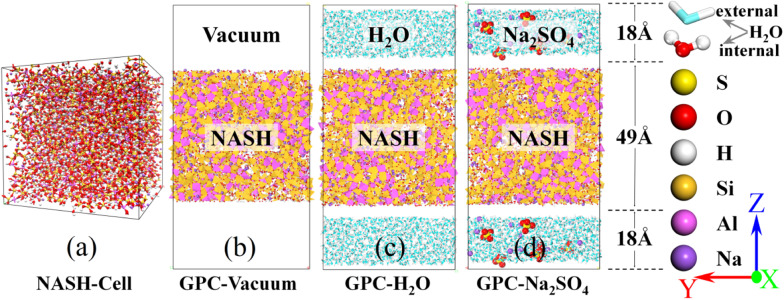
(a) is the constructed initial NASH cell. (b), (c) and (d) are the initial simulation models in vacuum, H_2_O, and Na_2_SO_4_ environments, respectively.

According to He *et al.*,^34^ the microstructure and mechanical properties of NASH were examined, revealing an increase in tensile strength and a slowdown in polymerization rate as the Si/Al ratio increased. In this simulation, we utilized the Si/Al ratio of 3.0 and Na/Al ratio of 1.0, which exhibited superior mechanical properties. Additionally, we constructed a NASH gel model based on the self-assembly concept proposed by Guan *et al.*,^25^ aiming to create a three-dimensional network structure. To match Si/Al = 3.0, we chose Mo4, Mo5, Mo6 and Mo7 building blocks (oligomers). A certain amount of oligomers and Na matching the ratios were placed in the simulation box and the build simulation was performed in LAMMPS software. To accelerate the rate at which the polymerization reaction occurs, we slowly increased the initial stacking model with a certain ratio from 300 K to 1800 K in 600 ps under the NVT system, and the Si–O and Al–O tetrahedra underwent a rapid dehydration and condensation reaction to form a gel-like structure. The high-temperature environment of 1800 K was continued to be maintained at a constant temperature of 500 ps to increase the reaction rate of the stacking model under the NVT system. This was followed by 200 ps of annealing treatment (NVT) and 300 ps of constant temperature reaction at 300 K (NPT), a process that would allow the geopolymer hydration products to form a stable gel structure to ensure the reproducibility and stability of the simulation. The dimensions of the final NASH initial model obtained were 42.71 × 42.68 × 44.22 Å^3^ and the microstructure is shown in Fig. 1(a). In our simulation, we employed a vacuum condition model to represent the drying conditions in actual engineering applications. This method has been widely adopted in numerous simulation studies^18,33,35,36^ to investigate similar problems. In experimental investigations of the impact of Na_2_SO_4_ solution on the concrete matrix, the concentration is typically chosen within the range of 0 to 15 wt%.^37–41^ Furthermore, the higher the content of Na_2_SO_4_, the more evident its effect on the matrix becomes. In our simulations, we selected a 10 wt% Na_2_SO_4_ solution^28,39,41,42^ as it not only demonstrates the exceptional resistance to sulfuric acid erosion of GCM but also ensures distinct differences in simulation results among different systems, facilitating the identification of patterns and analysis of phenomena. Finally, the NASH gel structure obtained on appeal was placed in vacuum, H_2_O, and 10 wt% Na_2_SO_4_ solutions, as shown in Fig. 1(b)–(d), and the simulation models of NASH in the three environments were constructed, which were used to compare the resistance to sulfate erosion and the mechanical properties of the NASH cementitious materials in different erosion environments. The entire simulation process was conducted on a 120-core workstation, with each model utilizing 60 cores for computation. The simulation process took approximately one week to complete.

### 2.3 Uniaxial tensile test

Uniaxial tensile simulations were conducted to investigate the mechanical properties of NASH under different erosion environments using the LAMMPS software.^43^ Throughout the tensile deformation MD simulation, the time step set to 0.25 fs and the temperature set to 300 K. Firstly, the erosion deterioration process is carried out for 1 ns under the NPT system, where the H_2_O molecules, SO_4_^2−^ and Na^+^ are continuously approach the substrate structure and intruding into the interior of the substrate to a certain extent, causing the matrix structure to change. Then, for the 3D reticulated NASH gel, which exhibits amorphous isotropic characteristics, we chose to reveal the mechanical properties of this material by stretching along the *Y* direction. In the relevant literature on uniaxial tensile simulations, the engineering strain rates during dynamic simulations are in the range of approximately 10^−5^–10^−2^ ps^−1^.^44–48^ Therefore, under the NVT system, we used a constant engineering strain rate of 0.001 ps^−1^ for uniaxial tensile deformation of the system. This means that the length increment of the analog box is constant for a certain time and the change of strain from 0 to 1 means that the length of the analog box in the *Y*-direction changes from the initial length to twice the length. Eventually, engineering stress–strain curves for different systems can be obtained, and the ultimate tensile strength and ultimate tensile strain can be determined from the peaks of these curves. These parameters will be used to characterize the state of change of tensile properties and structural toughness of the material in an erosive environment. The entire simulation process was conducted on a 120-core workstation, with each model utilizing 60 cores for computation. The simulation process took approximately one week to complete.

## 3 Results and discussions

### 3.1 Performance degradation analysis of NASH in erosive environments

**Fig. 2:**
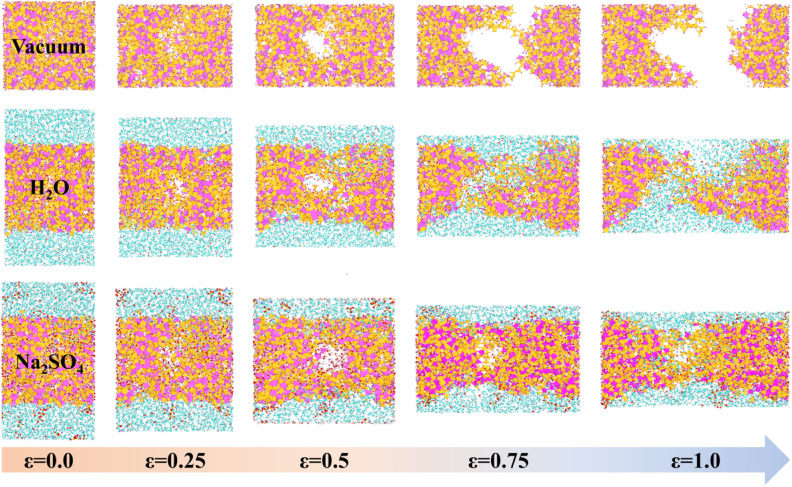
Snapshots of tensile fracture of NASH in different environments. Where vacuum, H_2_O, and Na_2_SO_4_ represent the simulated systems of NASH in vacuum, H_2_O, and sodium sulfate environments, respectively.

To observe the degradation characteristics of GPC materials in erosive environments, we initially conducted a *Y*-direction uniaxial tensile simulation on the model. The tensile fracture process of NASH in different environments is presented in Fig. 2. In both vacuum and H_2_O environments, NASH demonstrated distinct fracture occurrences at approximately 0.75 Å Å^−1^ and 1 Å Å^−1^, respectively. Comparatively, NASH displayed less apparent fracture during the tensile process in the Na_2_SO_4_ environment when compared to the previous two systems. These results indicate that NASH can effectively retard the structural degradation induced by tensile loading in H_2_O or Na_2_SO_4_ environments. Additionally, NASH exhibits increased durability under prolonged tensile loading in the presence of Na_2_SO_4_. This phenomenon can be attributed to the penetration of external solutions into NASH through cracks formed during the tensile process. Water or ions then permeate into the cracks, leading to matrix erosion while simultaneously establishing new interactions to mitigate the degradation caused by tensile loading. A comprehensive analysis detailing this phenomenon is provided in Section 3.2 of the article.

**Fig. 3:**
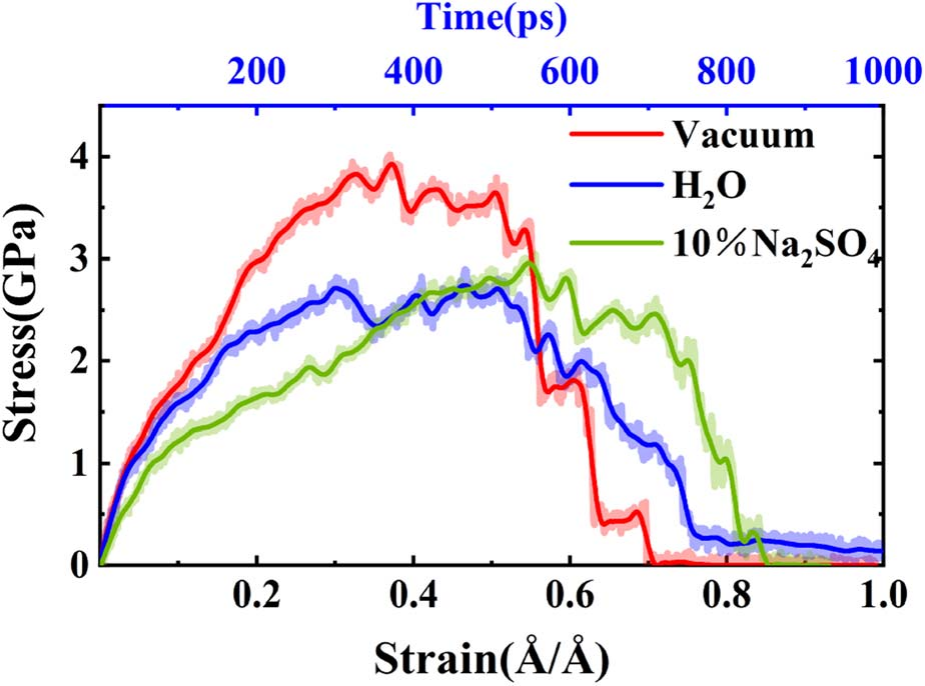
Stress–strain (stress–time) curves of NASH in different environments. Where the dashed line is the original data curve and the solid line is the curve fitted to the original data.

To further analyze the degradation process, we extracted the variations in stress and strain during the tensile fracture process, and the resulting stress–strain diagram is depicted in Fig. 3. Initially, we identified the stress value corresponding to the peak point on the stress–strain curve as the (ultimate) tensile strength of the material. As illustrated in Fig. 3, the tensile strengths of NASH in vacuum, H_2_O, and Na_2_SO_4_ environments were determined to be 3.93 GPa, 2.74 GPa, and 2.96 GPa, respectively. Consequently, compared to the vacuum environment, the tensile strength of NASH decreased by 30.28% and 24.68% in H_2_O and Na_2_SO_4_ environments, respectively. This observation suggests that the pure H_2_O environment exerts a more pronounced detrimental effect on the mechanical properties of the NASH system compared to the ionic Na_2_SO_4_ environment. This finding aligns with the existing literature highlighting the slight enhancement in the mechanical properties of GPC under Na_2_SO_4_ solution compared to an aqueous solution.^19–22,49–51^ Such information holds crucial importance for the design and performance assessment of engineering structures, including buildings and bridges.

Also in Fig. 3 the stress and strain *versus* time are given. Both the time taken to keep the matrix stable (strength) and the time taken to destroy the matrix (strength reduced to 0), the NASH matrix has a longer tolerance time in the Na_2_SO_4_ environment. Therefore, the dissolution of the NASH matrix is slower in the Na_2_SO_4_ environment and it has better resistance to sulfate attack. As shown in Fig. 3, the ultimate tensile strains of NASH in vacuum, H_2_O, and Na_2_SO_4_ environments are 0.37 Å Å^−1^, 0.47 Å Å^−1^, and 0.55 Å Å^−1^. Compared to the vacuum environment, the ultimate tensile strains of NASH in H_2_O and Na_2_SO_4_ environments are increased by 27.03% and 48.65%, respectively. Comparing the variations in tensile strength and ultimate tensile strain of the NASH matrix under different conditions, it can be concluded that, overall, the presence of a H_2_O environment has a detrimental effect on the performance of the NASH matrix. On the contrary, in the Na_2_SO_4_ environment, although the tensile strength decreased to a certain extent, the ultimate tensile strain changed greatly and increased almost by half, and overall the sulfate erosion resistance and tensile properties of NASH matrix were better than those of NASH matrix in the vacuum environment. This indicates that the material has better ductility and can undergo greater deformation in the Na_2_SO_4_ environment, and also responds to the increased resistance to crack extension and improved durability and toughness of the material.

The augmentation of the ultimate tensile strain contributes significantly to the enhancement of material availability and safety in specific conditions. Particularly, in the case of structural materials like prestressed concrete,^52^ despite the decrease in ultimate tensile strength observed in the Na_2_SO_4_ environment, the safety of the structure is compromised and the risk of structural damage due to tensile loading is increased to some extent. However, concurrently, the toughness is improved, resulting in a transition from brittle to ductile damage mode. Consequently, the resistance to crack propagation and rupture is improved. Additionally, the improved resistance to sulfate attack and overall toughness of NASH structures in Na_2_SO_4_ environments prove beneficial in specific applications, such as offshore structures,^53^ wherein greater deformation is required. Furthermore, in applications that necessitate highly ductile materials,^54^ particularly subject to impact or dynamic loading, toughness, and fracture modes are of paramount importance.

### 3.2 Microstructure analysis of NASH in erosive environments

#### 3.2.1 Solution erosion

**Fig. 4:**
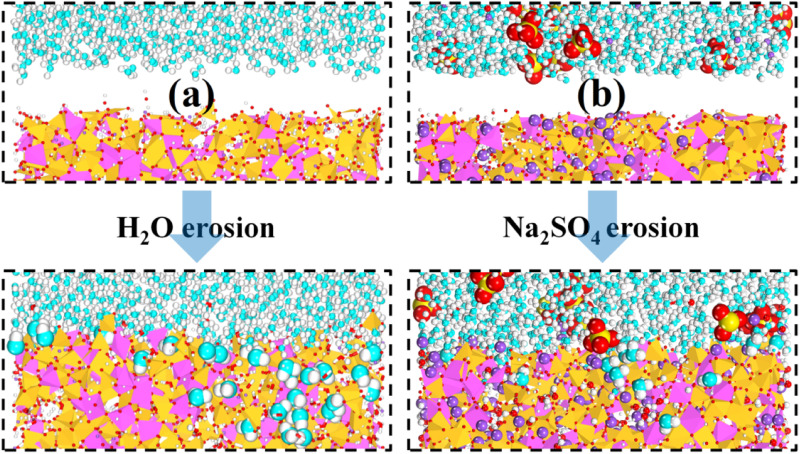
(a) and (b) show the local structure of the interface during the simulated equilibrium phase in H_2_O and Na_2_SO_4_ environments, respectively.

To investigate the microscopic mechanism leading to the above results, we first observed the deterioration state of the material in different environments, and the local structure of the simulated equilibrium stage is shown in Fig. 4. In Fig. 4(a) water molecules continuously approach the matrix and penetrate deep into the interior of the matrix after a static erosion of 1 ns. It is also observed that some water in the matrix also diffuses into the aqueous solution, and water substitution occurs. For the matrix structure, the NASH gradually becomes swollen from a relatively dense state, so it can be determined that the material undergoes some structural changes in the aqueous environment, which are more concentrated in the surface structure in contact with water and less in the interior.^18^ In the Na_2_SO_4_ environment, as shown in Fig. 4(b), the behavior of water molecules is similar to the above, while some of the SO_4_^2−^ ions are adsorbed to the surface of the substrate, and some of the Na^+^ ions enter into the interior of the substrate.

**Fig. 5:**
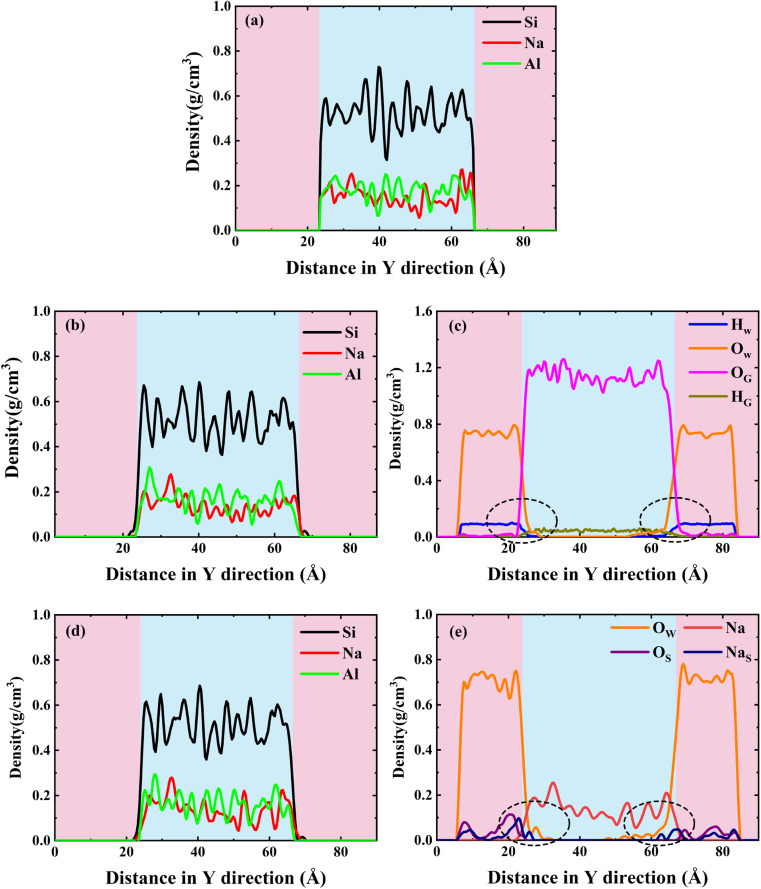
Density profiles of simulated equilibrium phases of the NASH system in (a) vacuum, (b and c) H_2_O, and (d and e) Na_2_SO_4_ environments. Where Si, Na, Al, O_G_, and H_G_ denote silicon atoms, sodium atoms, aluminum atoms, oxygen atoms in H_2_O, and hydrogen atoms in H_2_O in the NASH matrix, respectively; and O_W_, H_W_, O_S_, and Na_S_ denote oxygen atoms in H_2_O, hydrogen atoms in H_2_O, oxygen atoms in the sulfate ions, and sodium atoms in the solution in the external environment, respectively.

To better characterize this phenomenon, we plotted the density distribution as shown in Fig. 5. The middle of the density distribution diagram is the NASH matrix and the two sides are the external environment. As shown in Fig. 5(a), (b), and (d), the density distributions of the intermediate region do not vary much in different environments, indicating that the backbone of the NASH structure does not change much in H_2_O and Na_2_SO_4_ environments, but only the three-dimensional dimensions are altered. This is in general agreement with what has been reported in the literature.^28^ Since NASH is isotropic,^24,25^ there is little difference in the expansion in the three directions. In Fig. 5, the boundary between NASH and the external environment is established based on the position at which the density distribution of Al atoms approaches zero. The thickness of NASH is 43.125 Å in a vacuum environment, while it is 44.250 Å and 44.625 Å in H_2_O and Na_2_SO_4_ environments, respectively, with an increase in size of 2.61% and 3.48%, and an increase in volume of 8.03% and 10.80%. This is the reason for the decrease in tensile strength, where water molecules invade the matrix causing swelling, leading to an increase in the Al–O and Si–O bond lengths, a decrease in bond stability, and a disruption of the three-dimensional network structure.

It is also mentioned in the literature that water molecules mainly induce hydrolysis of Al–O bonds and hardly affect the stability of Si–O bonds, which is consistent with our analysis.^18^ Meanwhile, we also counted the difference in the average density of the NASH matrix in vacuum, H_2_O, and Na_2_SO_4_ environments, which were 2.04, 2.03, and 2.06 g cm^−3^, respectively, and the structure of the matrix in the Na_2_SO_4_ environment was more dense, and the increase in the compressive strength of NASH in Na_2_SO_4_ environment reported in the literature is the reason for the structural densification,^55^ which indicates that our simulation of the decrease in matrix tensile strength is plausible and valid. Subsequently, in Fig. 5(c), a noticeable exchange of water molecules occurs, primarily involving the infiltration of external water molecules into the matrix, alongside a minor diffusion of internal water molecules into the external environment. Moreover, the water molecules invade deeper and more in the Na_2_SO_4_ environment, together with the invasion of Na_S_ with a larger radius, which is the reason why the matrix swells more than in the H_2_O environment. The more water intrusion, the more volume expansion, and generally lower tensile properties.^56^ However, the previous study showed that the tensile properties were better in the Na_2_SO_4_ environment, which may be the reason for the intrusion of Na^+^ ions in the solution. As shown in Fig. 5(e), the intrusion of external Na^+^ ions lags behind the infiltration of external water molecules. This indicates that during the entire intrusion process, external water molecules first infiltrate the matrix and disrupt the Al–O bonds, resulting in chain breakage, expansion of the NASH matrix, and an increase in internal voids within the matrix. Subsequently, the larger atomic radius of Na^+^ allows it to be adsorbed into the NASH matrix by the adsorption sites within the matrix (due to the difference in electronegativity), thereby improving the relevant performance. The role of Na^+^ ions is described in Section 3.2.2.

#### 3.2.2 Interaction

**Fig. 6:**
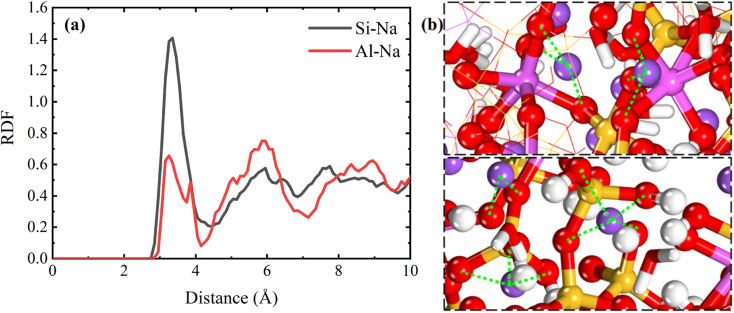
(a) RDF plot of NASH system after equilibration in simulated equilibrium phase in Na_2_SO_4_ environment. (b) Local snapshots of ligand formation after adsorption of external Na^+^ by adsorption sites during the simulated equilibrium phase (to facilitate statistical calculations, Si–Na is used to denote the interaction between Na⋯O–Si, below).

**Fig. 7:**
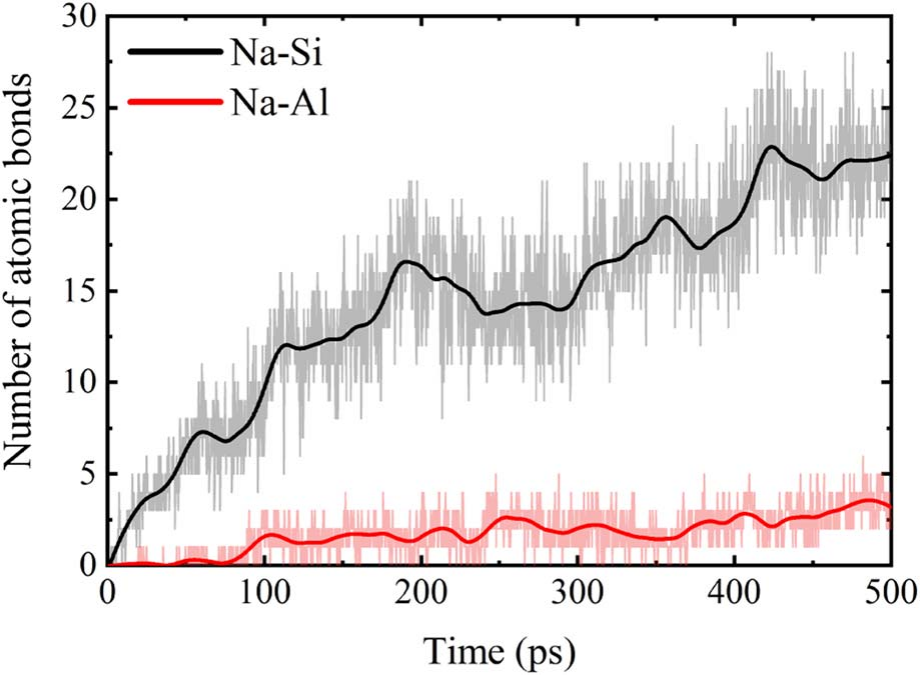
Number of Na coordination bonds formed by adsorption sites adsorbed throughout the simulated equilibrium phase of the NASH system in Na_2_SO_4_ environment.

The water molecules enter into the matrix and make the NASH become loose with many vacancies, and the water molecules and Na^+^ in the NASH have higher space and freedom, and the external Na^+^ ions can be adsorbed into the matrix by the “adsorption sites” in the NASH. As shown in the RDF diagram in Fig. 6(a), the external Na is embedded in SiO and AlO polyhedra, and Na and O are coordinated to form NaO polyhedra, where Na is firmly fixed. This adsorbed coordination state is also observed in the local snapshot image of the simulated equilibrium phase shown in Fig. 6(b). The number of Na⋯O–Al bonds and Na⋯O–Si bonds gradually increases with the extension of the equilibrium time, as shown in Fig. 7. Because of the relatively high Si content in the matrix, the external Na^+^ is mainly adsorbed by the SiO polyhedra, coupled with the fact that the Si–O bond is more stable than the Al–O bond under hydrolysis, the high number and relatively more stable Na⋯O–Si linkage structure is not easy to be destroyed by water molecules. As more external water molecules intrude into the NASH matrix, leading to the gradual expansion of the NASH matrix, it is easier for external Na^+^ to enter into the interior of the matrix, and the Na adsorbed by the AlO polyhedra only gradually increases. Although the swelling of the matrix will lead to a decrease in tensile strength, the expansion is limited, and Na^+^ and H_2_O are able to fill the vacancies and increase the number of internal hydrogen and ionic bonds, which makes the three-dimensional mesh structure of the matrix more complex and improves the tensile properties of NASH to a certain extent, which is consistent with the previous analysis. However, this does not explain the toughness change in the Na_2_SO_4_ environment well, so the structural evolution of the tensile fracture process is further analyzed.

#### 3.2.3 Chain structure

**Fig. 8:**
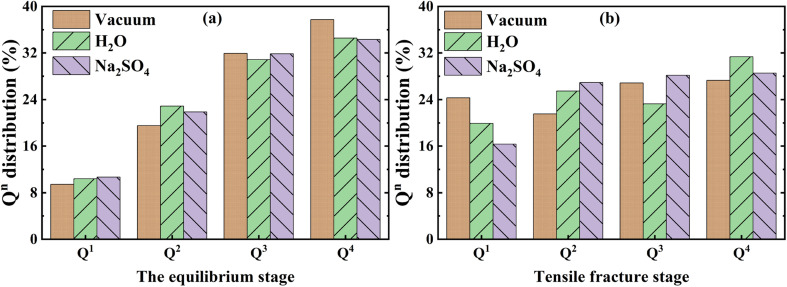
(a) and (b) show the variation of Q distribution in the equilibrium stage and tensile fracture stage respectively.

**Fig. 9:**
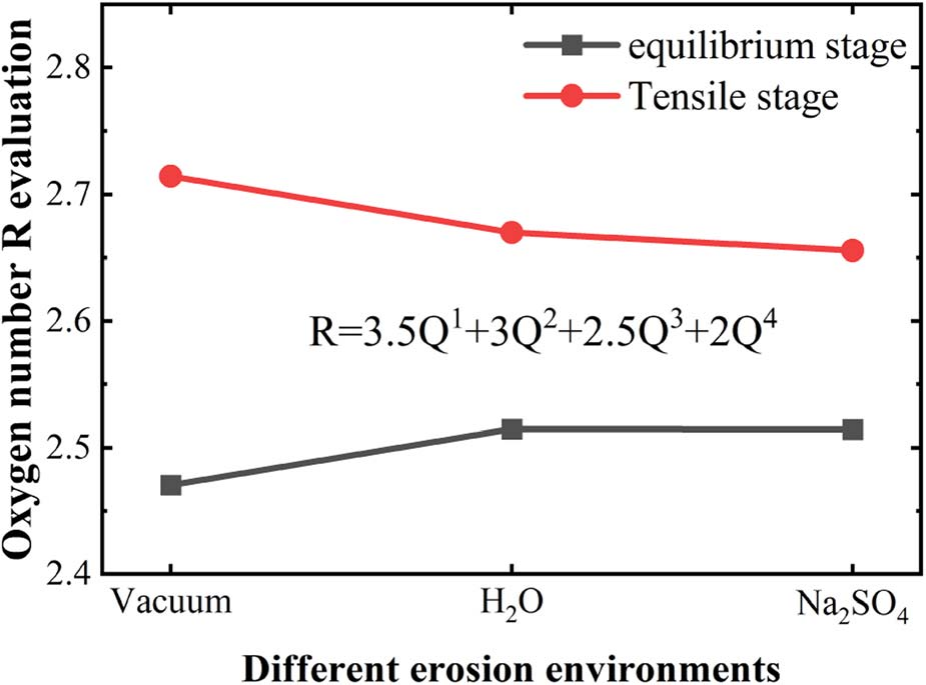
Plot of *R*-value variation of NASH structures at equilibrium stage and tensile fracture stage in different environments.

Next, a quantitative analysis of the changes in the material structure during the static and tensile erosion phases was carried out. The material structure affects the material properties, and the skeleton structure is particularly important in the whole structural system, the NASH gel skeleton structure is a three-dimensional network structure, and Q^*n*^ refers to the connection status between the Si–O and the Al–O tetrahedra, whereas the change in the content of Q^*n*^ can be used to characterize the damage of the skeleton structure. The change in Q^*n*^ content can be used to characterize the damage condition of the skeleton structure. The distribution of Q^*n*^ in the equilibrium stage is shown in Fig. 8(a). Compared with the vacuum environment, it is found that the contents of Q^1^ and Q^2^ increase while Q^3^ and Q^4^ decrease in H_2_O and Na_2_SO_4_ environment, which indicates that the intrusion of the external solution destroys the three-dimensional reticulated skeleton structure of NASH, which makes the Si–O bond or Al–O bond located in the bridge position broken and causes the reduction of the tensile strength. We also calculated the oxygen number (O/(Si + Al)) *R* of different systems, as shown in Fig. 9, and the value of *R* can characterize the degree of connectivity of the backbone structure; the smaller the value of *R*, the better the connectivity of the 3D network backbone structure. In general, as the *R*-value increases, the network structure will gradually change from shelf-like to layer-like and chain-like, or even to cluster-like and island-like. To simplify the calculations, coupled with the relatively more stable and numerous Si–O linkage structure, as well as the fact that AlO tetrahedra form AlO pentahedra or hexahedra after being destroyed by hydrolysis and destroying the backbone structure,^18^ it can be assumed that the *R*-values of only SiO and AlO tetrahedra can be considered to be representative of the degree of backbone connectivity of NASH as a whole. The *R*-values of the equilibrium stage in Fig. 9 in H_2_O and Na_2_SO_4_ environments are approximately equal, indicating that the degree of erosion of the NASH mesh structure in the two environments is equal, but because the intrusion of Na from the external environment compensates for the structural damage brought about by the H_2_O erosion to a certain extent, which is ultimately manifested in the fact that the tensile strength in the Na_2_SO_4_ environment is stronger than that in the H_2_O environment. This is consistent with the results of the previous analyses.

**Fig. 10:**
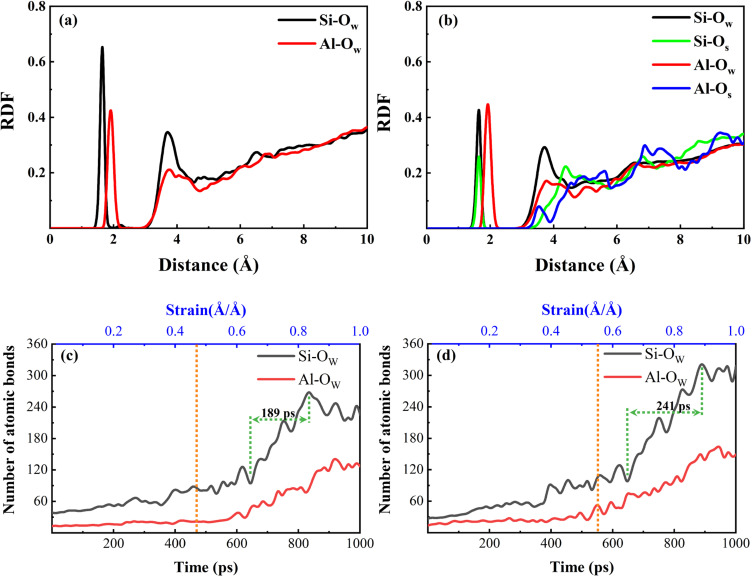
(a) and (b) RDF plots of the equilibrium stage in H_2_O and Na_2_SO_4_ environments, respectively; (c) and (d) plots of the number of atomic bonds *versus* stretching time for the tensile fracture stage in H_2_O and Na_2_SO_4_ environments, respectively.

**Fig. 11:**
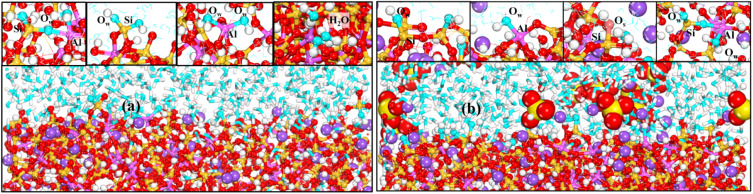
(a) and (b) show local snapshots of the equilibrium phase in H_2_O and Na_2_SO_4_ environments, respectively.

To have a clearer understanding of the structural damage caused by hydrolysis, we therefore made the following RDF plots. As shown in Fig. 10(a), the first peak of Si–O_W_ is higher in the equilibrium stage, which indicates that the external water is more easily attracted by Si in the aqueous environment, and the water molecules induced the Si–O bonds to break and hydrolyzed to form Si–OH, which destroys the three-dimensional network skeleton, and weakens the mechanical properties of the matrix. As shown in Fig. 10(b), the first peak of Al–O_W_ is higher, indicating that the external water is more easily attracted by Al in the Na_2_SO_4_ environment, inducing Al–O bond breaking to form Al–OH. The reason for this difference may be because the external SO_4_^2−^ ions are easily attracted by Si and Na, replacing a part of Si–O_W_ coordination, resulting in a relative decrease in the coordination of Si–O_W_. The validity of this explanation can also be confirmed by the fact that O_S_ only forms coordination with Si as seen in Fig. 10(b). It can also be observed in the local snapshots of the equilibrium stage as shown in Fig. 11 that the water molecules enter the matrix and partially hydrolyze to replace the unbridged oxygen of Si and Al, very few to replace the bridged oxygen, and most of them exist in the backbone interstitials in the form of free oxygen atoms. In Fig. 11(b) it is also observed that Na ions are adsorbed around Al–O and Si–O polyhedra to form coordination structures.

The change of Q^*n*^ distribution in the stretching stage is shown in Fig. 8(b), and we found that there is a significant increase in the Q^1^ content while the Q^2^, Q^3^, and Q^4^ contents all decrease to some extent in the pulling in a vacuum, which indicates that the mesh backbone structure is fractured and transformed into a low-polymerization state in the pulling process. Compared with the vacuum environment, NASH gel in a H_2_O environment, Q^1^, and Q^3^ content decreased while Q^2^ and Q^4^ increased, indicating that the presence of H_2_O to a certain extent slowed down the chain pulling process, this result is consistent with the increase in toughness of the previous complaint. Whereas, in the Na_2_SO_4_ environment, Q^1^ content decreased and Q^2^, Q^3^, and Q^4^ content increased compared to a vacuum environment, indicating that fewer monomers and more long and branched chains were produced during the pulling-off process, again slowing down the chain breakage and increasing the structural toughness. To better explain the difference in the strength of the H_2_O and Na_2_SO_4_ environments in slowing down the tensile degradation, we likewise calculated the oxygen number *R* of the different systems during the tensile stage, as shown in Fig. 9. It can be seen that in vacuum, H_2_O and Na_2_SO_4_ environments, the *R*-value of the system decreases sequentially, indicating that the Na_2_SO_4_ environment can slow down the tensile deterioration of the NASH structure to a greater extent, with a greater increase in toughness. Subsequently, we also counted the difference in the degree of hydrolysis during tensile fracture, as shown in Fig. 10(c) and (d). The dashed line in the figure indicates the time at which the system reaches the ultimate tensile strain. Before reaching the ultimate tensile strain, the NASH matrix exhibits a higher concentration of Si–O_W_ and Al–O_W_ bonds in the presence of a Na_2_SO_4_ environment. This suggests that the three-dimensional network framework of the matrix is reinforced during the tensile process due to the ingress of external Na^+^ ions, thereby enhancing the structural stability of the system and enabling it to withstand stronger dynamic loads. Additionally, by allowing more water molecules to infiltrate without disrupting the chain structure, the hydrogen bonding network within the matrix becomes more intricate, further strengthening the structure of the NASH matrix and delaying the tensile fracture process. On the other hand, this strengthening also slows down the hydrolysis process, which can be seen in the longer duration of the significant increase in the number of Si–O_W_ and Al–O_W_ bonds after reaching the ultimate tensile strain.

## 4 Conclusion

The study utilizes molecular dynamics research methodology to explore changes in performance and deterioration mechanisms of NASH in erosive environments. The following conclusions were drawn:

The tensile strength of NASH cementitious materials ranks as vacuum > Na_2_SO_4_ > H_2_O, while the ultimate tensile strain follows the sequence Na_2_SO_4_ > H_2_O > vacuum. Na_2_SO_4_ either strengthens or retards the deterioration of NASH cementitious materials rather than erosion. Additionally, its tensile fracture mode changes from brittle to ductile damage, enhancing the toughness and fracture resistance of the material.

In the Na_2_SO_4_ environment, water molecules invade the NASH matrix and undergo hydrolysis, leading to the destruction of the Al–O bond. Although the intrusion of water molecules also causes limited expansion of the matrix, it enlarges the internal vacancies within the NASH three-dimensional network skeleton and enhances the internal degrees of freedom.

Na^+^ ions from the external environment easily penetrate the matrix trailing behind the water molecules and are subsequently adsorbed onto the active oxygen adsorption sites on the “Si–O tetrahedra” or “Al–O polyhedral” within the matrix. These ions then undergo a coordination reaction, increasing the proportion of Q^2^, Q^3^, and Q^4^, reducing the *R*-value of the oxygen number, and reinforcing or repairing the three-dimensional network skeleton.

In the tensile fracture stage, Na^+^ ions combine with reactive oxygen species generated through internal stretching or induced by water molecules to repair the fractured skeleton. A reinforced three-dimensional network enhances toughness and retards the hydrolysis process. This results in the material's ability to withstand increased and prolonged tensile loads, thereby extending the time for the NASH gel material to reach complete fracture.

## Conflicts of interest

There are no conflicts to declare.

## Supplementary Material
